# How is parenting stress related to parental burnout among children’s mothers in China: the mediating role of marital satisfaction and the moderating role of socioeconomic status

**DOI:** 10.3389/fpubh.2024.1431598

**Published:** 2024-12-04

**Authors:** Xiaoyan Xu, Zahyah Hanafi, Shun Zhang

**Affiliations:** ^1^School of Education, Shandong Women’s University, Jinan, China; ^2^School of Education, Taylor’s University, Subang Jaya, Malaysia; ^3^School of Education, Faculty of Social Sciences and Leisure Management, Taylor’s University, Subang Jaya, Malaysia; ^4^School of Educational Science, Ludong University, Yantai, China

**Keywords:** parenting stress, parental burnout, marital satisfaction, socioeconomic status, mothers

## Abstract

**Introduction:**

Although parenting is a worthwhile and joyful process, it can also cause stress, potentially leading to parental burnout. With the implementation of the three-child policy in China, more parenting hours and higher economic costs may increase the risk of parental burnout.

**Objectives:**

This study investigated how was maternal parenting stress related to mothers’ parental burnout, as well as the potential mediating effect of their marital satisfaction and the moderating effect of their socioeconomic status on this relationship.

**Methods:**

Data were collected from 314 mothers living in mainland China. The Chinese versions of the Parenting Burnout Assessment Scale, Parenting Stress Index, and Marriage Perception Scale were used to measure mothers’ parental burnout, parenting stress, and marital satisfaction.

**Results:**

Mothers‘ parenting stress was significantly and positively related to mothers’ parenting burnout. Mothers’ marital satisfaction mediated this relationship. Mothers’ socioeconomic status moderated the first half of the mediation model, and parenting stress exhibited a greater effect on marital satisfaction when mothers had a higher socioeconomic status.

**Discussion:**

These findings indicated that mothers’ parenting stress could be alleviated by increasing marital satisfaction, which, in turn, reduced the risk of parental burnout. Furthermore, socioeconomic status may enhance the negative effects of parenting stress on marital satisfaction among mothers.

## 1 Introduction

Parenting burnout (PB) has been a hot topic in parenting research for the last decade (1–3). PB is an emotional disorder related to parenting that develops when a parent is exposed to parenting stress for an extended period (3). The main symptoms are feelings of extreme exhaustion concerning one’s parental role, emotional detachment from one’s children, and a sense of losing parental self-efficacy, all of which are markedly different from one’s own previous parenting experiences (4). Since a new measurement tool—Parental Burnout Assessment (PBA) was developed (4), and a theoretical framework for PB—The Balance Between Risks and Resources (BR^2^) was raised (5), PB was receiving growing attention and exploration.

There is an increasing focus on PB in various countries, with studies documenting that PB is prevalent in most countries around the world (e.g., Finland, Belgium, France, Netherlands, United Kingdom, Sweden, Japan) (1, 6–8). A 42-country study established that the incidence of PB is approximately 5% in Western countries and 1–2% in Asian countries including China (1). Contemporary parents have higher expectations of their parenting behaviors (9) and hope to play the perfect parent role (10), such as setting detailed plans and requirements for their children’s success, which may lead to anxiety and stress in parenting (11). Consequently, the risk of PB may rise (12). Such prevalence suggests that it is necessary to explore the factors that lead to PB.

### 1.1 Parental burnout among mothers in China

In China, with the implementation of the three-child policy, parenting is becoming a focus of public discussion in the community. Although parenting may create a great sense of worth and happiness in one’s life (13), it is not always easy or enjoyable and can sometimes be complex and even stressful (14). A more child means more hours spent parenting and economic costs which may exacerbate parenting stress. Increasing expectations, anxiety, and stress related to parenting have also been observed in China (15, 16). Thus, many Chinese parents may be at risk of PB.

Generally, the outcomes of PB were unfavorable. Burnout may cause parents to experience avoidance, suicidal ideation, drug addiction, alcoholism, sleep disorders, marital conflict, and feelings of alienation from their spouse (17, 18). Furthermore, parents experiencing burnout tend to be indifferent or even violent toward their children (18, 19), and perform other negative or extreme parenting practices such as psychological control, low-responsive parenting, and less-supportive parenting (20). Studies in China have also determined that higher paternal PB is associated with problem behaviors (21, 22) in children. To help enhance positive parenting experiences, this study examined the behind mechanism of PB in mothers in China.

The importance of investigating PB among mothers in China should be emphasized for several reasons. First, Chinese culture places considerable importance on family prosperity. As reflected in the traditional saying, “more children mean more happiness,” many Chinese families hope to have more children (23). Against the broad background of the state’s encouragement of childbearing, many young Chinese mothers feel pressured to have children. However, having another child can increase the risk of PB (19). Second, owing to the traditional division of labor in families, mothers play a major role in childrearing in China (24). However, in modern society, Chinese women have begun to develop their careers and the female employment rate in the country is even higher than in the West (25). Many working Chinese mothers must cope with the complex tasks of working and parenting simultaneously. If they do not have sufficient energy to take on the responsibility of parenting, stress, worry, and even burnout may occur. Previous research has found that PB manifests differently between fathers and mothers (26). Thus, the importance of mothers’ PB especially needs to be considered. Third, the antecedents and mechanisms of mothers’ PB in China remain unclear. Only a few studies addressed the effects of PB on both children and parents (27, 28). However, research on the potential correlates of PB has been conducted in other countries, such as France and Japan (12, 29). Thus, research on mothers’ PB in China is urgently needed.

### 1.2 Parenting stress and parental burnout

The definition of PB suggests that parenting stress is a critical risk factor for PB. The linkage between parenting stress and PB might be explained by Lazarus and Folkman’s stress theory. This theory indicates that a person will cognitively evaluate stressful events based on their resources. When stress is perceived as inescapable or exceeding one’s coping abilities, a range of psychological stress responses may develop, such as avoidance, fear, exhaustion, etc. (30). It can be seen that burnout is one of the psychological stress responses. A strong association between stress and burnout has been found in a variety of groups. For instance, social workers who experienced high levels of stress might have feelings of burnout (31). Physiotherapists’ job stress was positively related to burnout (32). Similarly, teachers’ work stress was a risk for occupational burnout in the United States (33) and China (34). The positive relationship between stress and burnout was found among graduate students (35). As to parents and the process of parenting, PB may be the result of parenting stress that has not been effectively dealt with. Parents may experience a range of psychological and behavioral stress responses, such as becoming bored with parenting, detaching themselves emotionally from their children, and feeling unable to cope with the responsibilities of parenting (4).

Furthermore, the conservation of resources theory argues that human beings actively seek, preserve, and maintain resources that are valuable to them. When individuals are faced with an actual or potential loss of a resource, they may experience negative psychological feelings (36). Parenting is a long-term, high-resource-demanding process. If a caregiver is constantly stressed in parenting, his/her resources may be under threat and consumption. Parents with a resource loss would tend to switch to defense mode to protect the resource from loss. They might experience avoidance, boredom and burnout reactions.

However, there was not enough exploration of the connections between parenting stress and PB. A study from Belgium demonstrated that parental stress is an indicator of parenting burnout during a COVID-19 epidemic (20). In China, the findings of the longitudinal study indicated that parenting stress was positively related to parental burnout in fathers (22). Nevertheless, whether this association is significant for Chinese mothers needs to be checked in this study. Based on these theories and reviews, the following hypothesis was established:

*Hypothesis 1*: Mothers’ parenting stress is positively related to mothers’ PB.

### 1.3 The Mediating role of marital satisfaction

This study raised that marital satisfaction was a critical factor which related to parenting stress and PB. Marital satisfaction is the subjective assessment of or attitude toward the relationship between a married couple (37). Couples experiencing excessive parenting stress may face greater emotional distress. According to the family stress model (38), emotional distress related to parenting can lead to increased conflict between couples and poor marital satisfaction. Referring to the spillover hypothesis, parenting stress may spill over into marital interactions (39). In a family system, each family member belongs to several subsystems (40). For example, a woman is both a mother and a wife. A mother’s experience of parenting and the spousal relationship are interdependent (41). Mothers’ negative feelings in parenting may “spill over” to poor satisfaction in marital relationships (42). Studies have reported a negative association between parenting stress and marital satisfaction (14, 43, 44).

Moreover, low marital satisfaction may be a risk factor for PB. BR^2^ theory suggests PB is not only associated with parenting stress. It addresses that PB is caused by a chronic imbalance between risks and resources (5). PB may emerge when the risks or demands of parenting outweigh parenting resources for a long time. Those factors that can add parenting distress such as low co-parenting and low family support can be called risk factors (5). Especially, studies have indicated that high spousal co-parenting was a protective factor that might prevent PB (21, 45, 46). Based on spillover theory (39), poor marital satisfaction may indicate poor spousal interactions, low co-parenting, and less spousal support (47). Therefore, poor marital satisfaction may exacerbate the risk of PB. Previous studies have shown that parents will experience less burnout when they are more satisfied with their marriage (12, 48). High marital satisfaction implies a close and favorable relationship between a child’s father and mother. Mothers with high marital satisfaction may obtain emotional support and parenting cooperation from their spouses, thereby easing their worries and responsibilities related to parenting and reducing the risk of PB (49). Thus, high marital satisfaction is a protective resource that balances the risk of parenting stress. The possibility of PB is decreased consequently.

Collectively, high parenting stress is related to low marital satisfaction, in turn, low marital satisfaction might increase PB. These results suggest that parenting stress affects PB through marital satisfaction. Therefore, the following is proposed:

*Hypothesis 2*: Mothers’ marital satisfaction mediates the relationship between mothers’ parenting stress and PB.

### 1.4 The moderating role of socioeconomic status

It is worth noting that parents’ socioeconomic status (SES, usually measured by family income and parents’ education level) is a crucial factor in family dynamics. SES is also found to be closely related to parenting beliefs or behaviors (50). Furthermore, the family stress model posits that family economic stress can directly lead to parental emotional stress, which, in turn, results in poor parenting (38). Families with high SES have greater access to financial, social, and human capital, and more positive parenting experiences than those with low SES (51, 52). Unsurprisingly, mothers with low SES are at greater risk of developing high levels of parenting stress (53, 54).

Moreover, evidence has indicated an association between SES and marital satisfaction (55), with researchers confirming a link between SES and marital satisfaction across cultures (56). If a mother with low SES, cannot meet the basic needs, she may focus less on intimacy with her partner (57). Some findings report a link between SES and PB. According to health-root-cause theory (58), SES is the primary factor influencing mental health. The resources gained through education and income improve mental status by influencing individual behavior and preferences (59). When mothers have access to resources because of their high SES, they may be more likely to avoid the risk of PB. Furthermore, in 2021, the Chinese government issued the “double-reduction” policy that aims to reduce the burden of homework and training beyond schooling for students (60). Chinese society is increasingly advocating for the promotion of children’s all-round physical and mental development, including the development of morality, intelligence, physical fitness, and aesthetics. Recent national policies have shifted the focus away from academically-orientated parenting. Parents must invest more financial resources and knowledge to improve their children’s overall development. Consequently, the role of SES in parenting has become increasingly pronounced, which may exacerbate the parenting pressures faced by mothers with low SES. However, little research has been conducted on the effects of SES on the relationships among parenting stress, marital satisfaction, and PB in China. Thus, based on the above literature review, the following is proposed:

*Hypothesis 3*: Mothers’ SES moderates the relationships among mothers’ parenting stress, marital satisfaction, and PB.

### 1.5 The Current study

**Figure 1:**
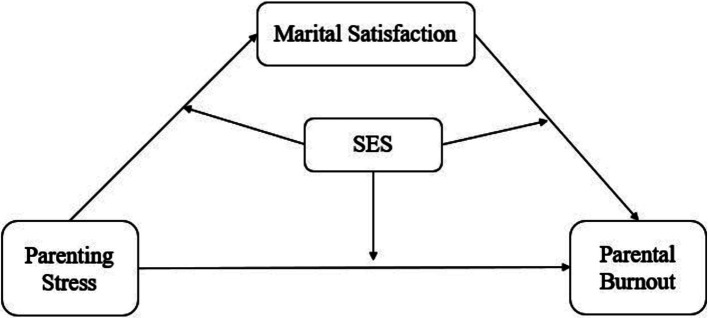
Research conceptual framework.

This study has the following three research objectives: (1) to examine the relationship between parenting stress and PB among mothers in China, (2) to explore the mediating effect of marital satisfaction on this association, and (3) to identify the moderating role of mothers’ SES in the mediating model Hypotheses 1, 2, and 3 correspond to research objectives 1, 2, and 3, respectively. The conceptual framework of this study is depicted in Figure 1.

## 2 Method

### 2.1 Participants

Data were collected online in February 2024 based on convenience sampling. Mothers of children aged 1–12 years were recruited through internet posters and friend referrals. To exclude the effect of postpartum depression, mothers of children under 1 year of age were not included. A total of 350 mothers completed an online survey and 314 questionnaires were valid (response rate of 89.71%). Most of the mothers were from East and South China (Shandong, Hebei, Shanxi, Jiangsu, Henan, Guangdong, etc.). Particularly, about one-third of the respondents were from Shandong.

Mothers raising more than one child were asked to select a child under the age of 12 for assessment. Demographic factors such as children’s age, gender, and the number of siblings (s) were collected according to a previous study (3, 4). Mothers’ children aged 1 ~ 2 was 58 (18.5%), children’s age range was 2 ~ 3 for 75 (23.9%), age range was 3 ~ 6 for 80 (25.5%), and age range was 6 ~ 12 for 101 (32.1%). 176 (56.1%) children were boys and 138 (43.9%) were girls. In addition, 173 (55.1%) mothers had one child, 128 (40.8%) mothers had two children, and 13 (4.1%) mothers had three or more children. The background information of mothers was as follows. The age of mothers was divided into five groups: below 25 years old, 26 ~ 30 years old, 31 ~ 35 years old, 36 ~ 40 years old, and above 40 years old. The percentages were 10.8% (34), 35.0% (110), 32.5% (102), 14.0% (44), and 7.6% (24), respectively. A total of 224 mothers (71.3%) were in full-time employment. 54 mothers (17.2%) were in part-time employment. While 36 mothers (11.5%) were unemployed. A total of 87 mothers (27.7%) came from rural and 277 mothers (72.3%) were from urban.

### 2.2 Procedures

This study was approved by the Academic Committee of Shandong Women’s University and conducted in accordance with the ethical standards outlined in the 1964 Declaration of Helsinki and its later amendments. Respondents were recruited through online advertisements and referrals from friends of participants. All the participants provided informed consent. Participants’ mothers read the instructions and information sheets provided online that explained the research objectives and fundamental principles (e.g., anonymity, no-harm, and confidentiality) and were informed that their participation was voluntary and could withdraw at any time. After the mothers provided their consent, they were instructed to answer the survey questions. This process required approximately 10 min to complete.

### 2.3 Measures

#### 2.3.1 Mothers’ parenting stress

Mothers’ parenting stress was assessed by using the revised Chinese version of the Parenting Stress Index (61) (e.g., “Since having this child, I can barely do the things I love.”). The 36-item scale includes three dimensions: parenting distress, dysfunctional parent–child interaction, and difficult child. [A five-point scale, was used with 1–5 indicating (1 = “strongly disagree,” 2 = “disagree,” 3 = “not sure,” 4 = “agree,” and 5 = “strongly agree,”)]. The mean total score was then calculated. A higher score indicated higher parenting stress. Scores ranged from 1 to 5, and Cronbach’s alpha of the scale and three dimensions were 0.96, 0.91, 0.95, and 0.93 in this study. The confirmatory factor analysis (CFA) was conducted to check the three-dimensional model by Amos 28.0. The modified model fit was as follows: *χ*^2^/*df* = 2.106, GFI = 0.822, NFI = 0.866, TLI = 0.945, CFI = 0.924, SRMR = 0.050, and RMSEA = 0.059, indicating that the construct validity of the scale was acceptable.

#### 2.3.2 Mothers’ marital satisfaction

Mothers’ marital satisfaction was measured by using the Chinese version of the Marriage Perception Scale (MPS) (62). The scale consists of 20 questions (e.g., “In general, I am very satisfied with my marriage.”) measured on a 7–point scale (1 = completely disagree, 7 = completely agree). There are three dimensions: couple interaction, couple relations, and couple conflicts. The mean total score was calculated. A higher score indicated more positive subjective feelings about marriage, implying higher levels of marital satisfaction. Scores ranged from 2.40 to 6.85 in this study. Cronbach’s alpha of the scale and three dimensions were 0.90, 0.91, 0.86, and 0.83. The modified model fit from CFA was as follows: *χ*^2^/*df* = 2.081, GFI = 0.904, NFI = 0.919, TLI = 0.947, CFI = 0.956, SRMR = 0.026, and RMSEA = 0.059, indicating that the construct validity of the scale was good.

#### 2.3.3 Mothers’ parental burnout

Mothers’ parental burnout was assessed by using the Chinese version of the Parental Burnout Assessment (PBA) (48). This is a 21–item, unidimensional scale. All the items referred to general parenting (e.g., “I feel completely run down by my role as a mother”). A 7–point scale was used, ranging from “1 = never,” “2 = several times a year,” “3 = once a month or less,” “4 = several times a month,” “4 = few times a month,” “5 = few times a month,” “5 = Once a week,” “6 = Few times a week,” and “7 = Daily.” The mean total score was the value used in the statistical analysis of this study. Higher scores indicate higher levels of burnout. Scores ranged from 1 to 7 in this study. In the present study, Cronbach’s alpha of the scale was 0.98. The modified model fit from CFA was as follows: χ^2^/*df* = 2.826, GFI = 0.876, NFI = 0.943, TLI = 0.951, CFI = 0.962, SRMR = 0.026, and RMSEA = 0.076, indicating that the construct validity of the scale was acceptable.

#### 2.3.4 Mothers’ Socioeconomic status (SES)

The *indicators of the SES* of mothers were measured by maternal level of education and family income. Previous studies have commonly used parental education and family income as the two key SES indicators (63). Considering that parenting is rooted in the family’s background, the mothers’ parenting will be based on the family’s general economic situation. Therefore, maternal economic status was measured using monthly family income. Mothers’ education level was categorized as “middle school complete and below,” “high school,” “college,” “university,” and “post-graduate (Master’s, PhD or an equivalent),” with each of choice assigned a value of 1 to 5. The percentages of the five education levels were 11.1, 18.2, 20.7, 43.6, and 6.4%, respectively. According to the National Bureau of Statistics (64), the *per capita* monthly disposable income is 3,268 RMB/person. According to Shandong Provincial Bureau of Statistics (65), the *per capita* monthly disposable income is 3,130 RMB/person. There are usually 1–2 children in a Chinese family. Accordingly, the average monthly income of a Chinese family may be around 10,000 to 12,000 RMB. Monthly family income was scored on an 8-point Likert scale (RMB): “5,000 and below,” “5,001–8,000,” “8,001–12,000,” “12,001–15,000,” “15,001–20,000,” “20,001–30,000,” “30,001–50,000,” and “50,000 or above,” which were assigned a value of 1 to 8. The percentages reported were 15.5%, 23.9%, 30.3%, 13.4%, 9.6%, 6.7%, 0.6%, and 0.6%, respectively. The largest percentage is the third level “8,001 to 12,000”. This is roughly in line with the data from the Bureau of Statistics. These two scores of education level and monthly income were then converted into *Z*-scores and added to obtain the SES score, higher indicated higher SES. Mothers’ SES ranged from −3.11 to 4.21.

### 2.4 Data analysis

Data were analyzed by using SPSS27 and AMOS 28.0. First, common method bias analysis was performed. As mothers answered the questions for all variables in this study, common method bias may have been present. Harman’s one-way test was used to examine this, setting the number of common factors to 1. The first principal component explained 35.935% of the total variance, which was less than the critical criterion of 40%, thus indicating no serious common method bias. Second, descriptive statistics and Pearson correlation coefficients were calculated for the main variables. The main variables, parenting stress, marital satisfaction, PB, and SES of mothers were tested for normality. The Skewness and Kurtosis of the variable distribution were examined. According to Kline, when the absolute value of Skewness is greater than 3 or Kurtosis is greater than 8, the distribution is considered severely non-normal (66). The test of normality revealed that the Skewness and Kurtosis values were within acceptable limits for all variables. Then the following Pearson correlation analysis and regression analysis could be conducted. Third, the PROCESS macro for SPSS (Model 4) was used to examine the mediating effect of mothers’ marital satisfaction on the relationship between parenting stress and PB. In addition, the PROCESS macro for SPSS (Model 59) was used to investigate the moderating effect of SES in that mediating model (67). All continuous variables performed in the models were standardized. The bias-corrected bootstrapping test based on 5,000 samplings was employed in mediation models. The 95% confidence interval which excludes zero is considered a significant effect.

## 3 Results

### 3.1 Preliminary analysis

The descriptive statistics and Pearson correlation coefficients are shown in Table 1. There was a significant positive correlation between mothers’ parenting stress and PB (*r* = 0.692, *p* < 0.001). Mothers’ marital satisfaction was negatively correlated to parenting stress (*r* = −0.253, *p* < 0.001) and PB (*r* = −0.369, *p* < 0.001). Mothers’ SES was negatively correlated with parenting stress (*r* = −0.149, *p* < 0.01) and positively correlated with marital satisfaction (*r* = 0.139, *p* < 0.05). The child’s age was correlated with the mothers’ PB (*r* = −0.172, *p* < 0.01) and parenting stress (*r* = −0.274, *p* < 0.01) negatively. Thus, the child’s age was included in the following model as a covariate.

**Table 1:** Descriptive and correlational analyses.

Variable	M ± SD	1	2	3	4
1 Mothers’ PS	2.54 ± 0.84	1			
2 Mothers’ MS	5.27 ± 0.89	−0.253^***^	1		
3 Mothers’ PB	2.79 ± 1.51	0.692^***^	−0.369^***^	1	
4 Mothers’ SES	0.06 ± 1.52	−0.149^**^	0.139^*^	−0.086	1
5 Child’s Age	–	−0.274^***^	0.008	−0.172_**_	−0.058
6 Child’s Gender	–	−0.111^*^	0.036	−0.071	−0.089
7 Number of Sibling (s)	–	−0.046	−0.126*	0.036	−0.094

PS is for parenting stress, MS is for marital satisfaction, PB is for parental burnout. ***p < 0.001, **p < 0.01, *p < 0.05.

### 3.2 Testing the mediating effect

Multicollinearity tests using Hierarchical Regression Analysis indicated acceptable variance inflation factors (VIF) below 3.0 (1.09 ~ 1.36), suggesting no serious multicollinearity problems. Mediation Model 4 of the SPSS macro prepared by Hayes was used to perform mediation effects analysis (67), controlling for the child’s age. The independent variable “mothers’ parenting stress,” the mediating variable “mothers’ marital satisfaction,” and the dependent variable “mothers’ PB” were centered. As indicated in Table 2, mothers’ parenting stress was positively associated with PB (*β* = 0.641, *p* < 0.001), thereby supporting Hypothesis 1. Mothers’ parenting stress was negatively related to marital satisfaction (*β* = −0.271, *p* < 0.001), and mothers’ marital satisfaction was positively related to d maternal PB (*β* = −0.207, *p* < 0.001). The mediating effect of mothers’ marital satisfaction was significant (*β* = 0.101, *p* < 0.001). The 95% confidence interval [0.049, 0.169] excluded 0. Thus, Hypothesis 2 was supported.

**Table 2:** Summary of regression models for parenting stress, marital satisfaction, and parental burnout of mothers.

Independent variables	Mothers’ MS			Mothers’ PB		
	*β*	SE	*t*	*β*	SE	*t*
Mothers’ PS	−0.271^***^	0.061	−4.753	0.641^***^	0.077	15.087
Mothers’ MS				−0.207^***^	0.069	−5.055
Child’s age	−0.067	0.046	−1.170	0.006	0.056	0.135
*R* ^2^	0.068			0.519		
*F*	11.303^***^			111.074^***^		

PS is for parenting stress, MS is for marital satisfaction, PB is for parental burnout. ***p < 0.001, **p < 0.01, *p < 0.05.

### 3.3 Testing the moderated mediating effect

**Figure 2:**
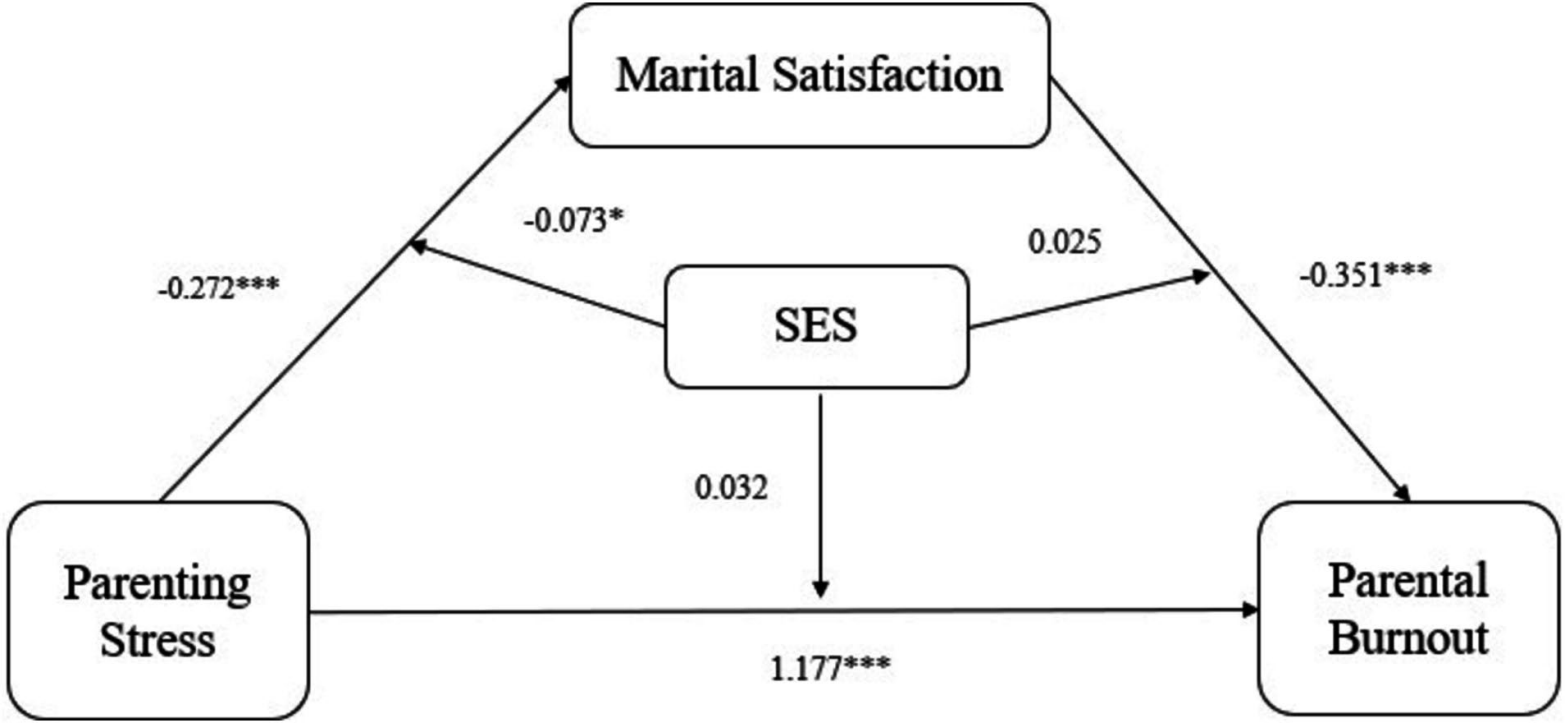
Test results of the mediation model with moderation. Control variable is Child’s age. ***p < 0.001, **p < 0.01, *p < 0.05.

A moderated mediating effects analysis was conducted using PROCESS Model 59, with the child’s age as the control variable. Simple slope tests were also conducted to examine the predictive effect across different SES levels (M ± 1SD) (68). As reported in Figure 2, Table 3, maternal parenting stress is positively related to PB (*β* = 1.177, *p* < 0.001). However, the interaction term between mothers’ parenting stress and SES was not significant in relation to PB (*β* = 0.032, *p* = 0.64). Therefore, mothers’ SES, did not moderate the association between parenting stress and PB.

**Table 3:** Summary of regression models for parenting stress, marital satisfaction, SES, and parental burnout of mothers.

Independent variables	Mothers’ MS			Mothers’ PB		
	*β*	SE	*t*	*β*	SE	*t*
Mothers’ PS	−0.272^***^	0.061	−4.469	1.177^***^	0.079	14.974
Mothers’ SES	0.051	0.032	1.566	0.039	0.041	0.952
Mothers’ PS × SES	−0.073^*^	0.035	−2.09	0.032	0.046	0.693
Mothers’ MS	–	–	–	−0.351^***^	0.071	−4.961
Mothers’ MS × SES	–	–	–	0.025	0.047	0.534
Child’s age	−0.040	0.046	−0.870	0.013	0.057	0.220
*R* ^2^	0.09			0.521		
*F*	7.611^***^			55.542^***^		

PS is for parenting stress, MS is for marital satisfaction, PB is for parental burnout, SES is for socioeconomic status. ***p < 0.001, **p < 0.01, *p < 0.05.

**Figure 3:**
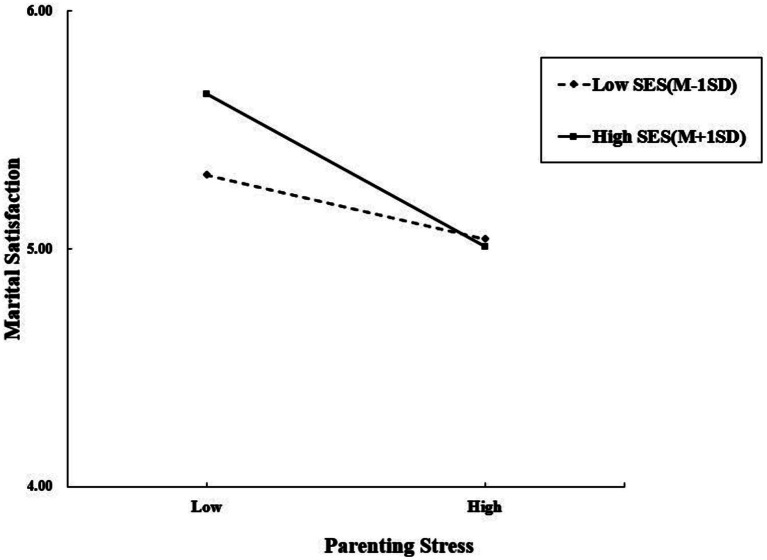
Simple slope analysis. SES is for socioeconomic status.

Table 3 and Figure 2 reveal that mothers’ parenting stress was negatively related to marital satisfaction (*β* = −0.272, *p* < 0.001), and the interaction term (mothers’ parenting stress × SES) was significantly related to maternal marital satisfaction (*β* = −0.073, *p* < 0.05). Thus, the relationship between mothers’ parenting stress and marital satisfaction was moderated by SES. As shown in Figure 3, for mothers of low SES, the positive association between mothers’ parenting stress and marital satisfaction (*β*_simple_ = −0.161, *p* > 0.05) was weaker than for mothers of high SES (*β*_simple_ = −0.384, *p* < 0.001).

Table 3 and Figure 2 show that mothers’ marital satisfaction was negatively related to parental burnout (*β* = −0.351, *p* < 0.001), but the interaction term (mothers’ marital satisfaction × SES) was not significantly related to PB (*β* = 0.025, *p* = 0.70). Thus, mothers’ SES did not moderate the relationship between mothers’ marital satisfaction and PB.

Table 4 indicated that for mothers with low SES, the indirect effect of parenting stress through marital satisfaction on PB was insignificant (*β* = 0.063, *p* > 0.05), with 95% confidence intervals including 0. For mothers with high SES, the indirect effect of parenting stress through marital satisfaction on parental burnout was significant (*β* = 0.120, *p* < 0.001), with 95% confidence intervals excluding 0. Therefore, Hypothesis 3 was supported partially.

**Table 4:** Results of the conditional indirect effect.

Pathway				SES	*β*	BootSE	Boot95% CI	
							Lower	Upper
Indirect effects of MS	Low	0.063	0.041	−0.004	0.160
	High	0.120***	0.052	0.031	0.232

MS is for marital satisfaction, SES is for socioeconomic status. ***p < 0.001.

## 4 Discussion

### 4.1 Main findings

This study explored the relationship between mothers’ parenting stress, marital satisfaction, PB, and SES in China. The findings suggest that mothers’ parenting stress has a positive effect on PB. Furthermore, this relationship was mediated by maternal marital satisfaction, while mothers’ SES moderated the relationship between parenting stress and marital satisfaction. The higher the mother’s SES, the stronger the effect of parenting stress on marital satisfaction. This moderating mediation model has significant potential in terms of developing targeted prevention and intervention programs for PB among mothers in China.

First, the findings revealed a positive relationship between mothers’ parenting stress and PB. Thus, Hypothesis 1 was supported. This was consistent with the significant association between Chinese fathers’ parenting stress and PB (22). These viewpoints follow Lazarus and Folkman’s stress theory which posits that excessive and persistent psychological stress with which an individual is unable to cope leads to a range of adverse physical and psychological reactions, such as mood disorders (69). Simultaneously, minor disturbances in daily life can have significant effects on individuals’ physical and mental health over time, thereby contributing to depression, anxiety, cardiovascular disease, and other negative consequences (70). Parenting is a lengthy and tedious process. Constant parenting stress can easily trigger excessive and persistent stress reactions, such as depression, anxiety, feelings of ineffectiveness, and emotional exhaustion. Moreover, over 70% of the mothers in this study were employed full-time. Thus, they not only needed to deal with the work pressures but also had to bear most of the childcare tasks in their household. The fourth survey on the social status of Chinese women revealed that mothers were primarily responsible for the daily caring and homework tutoring of children aged 0–17 years, and women spent 136 min a day taking care of children and other family members (71). It can be challenging for mothers with limited energy to simultaneously handle the dual stress of working and parenting. A small number of mothers in this study were either working part-time or parenting full-time. However, this does not imply that participants did not experience parenting stress. By contrast, they may give up their careers because no one in the family was available to share parenting tasks. A previous survey comparing the employment rates of two groups of urban mothers aged 25–34 years found that the percentage of mothers with children under 6 years of age was 72.0%, while that of mothers without young children was 10.9% higher (72). Thus, some women may choose to leave their jobs to take on the majority of household labor and childcare responsibilities because they are unable to balance childcare and work. When mothers feel the pressure of both work and family or when sharing parenting responsibilities with other family members is difficult, parenting risks will exceed the available resources (5). Parents may also experience parenting distress or emotional exhaustion, which may lead to a tendency to show more intense psychological aggression toward children, such as yelling (73). These psychological and behavioral manifestations are precisely what can be described as PB.

Second, results indicated that mothers’ parenting stress was not only directly related to PB, but also was indirectly related to PB through marital satisfaction. Thus, Hypothesis 2 was supported. The more stress, mothers experienced during parenting, the more likely it was that marital satisfaction would decrease, which, in turn, would lead to PB. These research findings echo family systems theory and the spillover hypothesis (39, 40). The borders of the parenting and marital subsystems are interpenetrating in the family system. Family members’ emotional experiences in one subsystem may spill over into the other. To be specific, the motherhood experience and the wifehood experience are interpenetrated. A mother’s parenting distress may cause her to be emotionally upset, and these negative feelings may overflow into the interactions between wife and husband. Mothers of children may show poor emotional responses to their husbands, which may lead to negative spousal relationships. As a result, the mother’s perceived marital satisfaction may be reduced. While, in accordance with the BR^2^ parenting theory (5), low marital satisfaction may be a dangerous factor for PB. The reason is that low marital satisfaction implies a poor husband-wife relationship. Mothers may have lower access to parenting resources such as spousal support, co-parenting, and so on. Mothers may develop PB if they chronically lack the necessary resources such as fathers’ co-parenting to handle parenting-related stressors (46). Conversely, in an excellent marital relationship, couples support each other in coping with challenges during childrearing, thereby providing parenting resources that mitigate the risks associated with parenting stress for mothers (12, 48). This study’s findings, which indicated that high marital satisfaction buffers parenting burnout, validate this theoretical view and provide direct evidence to support the BR^2^ parenting theory. This suggests that the theory not only explains PB well in Western cultures but is also equally applicable to Chinese contexts.

Third, this study found mothers’ SES exerted a moderating effect in the first half of the mediation model. Thus, Hypothesis 3 was partially supported. Mothers’ SES moderated the association between parenting stress and marital satisfaction, but not between parenting stress and PB, or marital satisfaction and PB. Specifically, maternal parenting stress was significantly related to mothers’ PB through marital satisfaction only among mothers with high SES. China is in a period of rapid urbanization, and social and economic development has brought about consumerism, materialism, and changes in life patterns and concepts of marriage (43). The results of this study indicate that SES is significantly related to parenting stress and marital satisfaction. This finding echoes the salient effect of SES on parenting and marriage. As seen in the simple slope analysis plot, mothers with higher SES are more likely to experience marital satisfaction than those with low SES when facing a similar level of parenting stress. Several reasons may explain this finding. First, high income can relieve the financial stress of parenting, thereby resulting in fewer couple conflicts. Second, higher levels of education mean that mothers are more knowledgeable and literate and can find help with scientific childrearing, thus reducing the pressure of the “double-reduction” policy. Third, employment may provide social support, feelings of self-worth, and independence for young working mothers. These positive cognitive and emotional experiences, in turn, have a favorable impact on parenting and marriage. Therefore, mothers with high SES are more likely to get support from their spouses when they face parenting stress (74), which decreases the influence of parenting stress and prevents PB.

Moreover, this study indicated that mothers’ SES not significantly associated with mothers’ PB. This is a similar conclusion to that of a previous study, in which the results provided little support for the direct effect of family SES on PB (75). In addition, mothers’ SES did not moderate the relationship between parenting stress and PB in the current study. In other words, the link between parenting stress and PB may be relatively stable and not influenced by the mother’s SES. These results suggest that objective resources such as high SES are not necessarily a resource against the risk of PB, but that subjective psychological resources such as marital satisfaction are more critical parenting resources.

### 4.2 Implications

On a theoretical level, by examining the relationship between maternal parenting stress, marital satisfaction, and PB and the moderating effect of SES on this relationship, this study deepens the research on the mechanisms of parenting burnout among Chinese mothers. Furthermore, these findings may contribute to the current understanding of the patterns of PB. From a practical perspective, this study’s findings can provide targeted guidance for the prevention of and interventions for mothers’ PB in China. The current study’s findings implied that increasing mothers’ psychological resources, such as by improving marital satisfaction, might be effective in alleviating negative parenting experiences and behaviors, which undoubtedly provides a practical pathway to promote positive parenting and improve mental health among mothers in China. Although real-life stressors such as raising young children are sometimes objectively difficult to escape, parenting risks can be balanced. When mothers feel emotionally supported by their partner and experience high marital satisfaction, negative parenting consequences such as PB may be prevented.

### 4.3 Limitations

Admittedly, this study has the following limitations. First, this study is only a preliminary exploration by a small sample size, the representativeness of the sample needs to be improved. The sampling method and size of the sample in this study were not sufficiently representative of the national group of mothers. In addition, respondents did not include mothers of children with special needs. Future research requires a nationwide large, multilevel, multiclass, and multicategory sample to explore the characteristics of PB more effectively. Investigations into other cultures should also be considered because all the participants were Chinese mothers.

Second, the study was limited to mothers. Although studies have examined the association between parenting stress and PB in fathers (22), whether the mechanisms behind it are consistent with mothers is almost unexplored. From parenting stress to PB, mothers and fathers may go through different processes. There may be two paths for mothers from parenting stress to PB. One is “maternal parenting stress to maternal psychological distress to poor marital relationships to less co-parenting to maternal PB” (44, 45, 47). The other may be “maternal parenting stress to low marital satisfaction to less co-parenting to maternal PB” (44, 45, 47). Yet there may be only one path from parenting stress to PB for fathers (44, 45, 47), similar to the second path for mothers. Future research needs to consider further exploring the mechanisms by which paternal parenting burnout occurs. At the same time, the effects of paternal co-parenting are necessary to examine in future studies. Furthermore, parenting is a joint endeavor of both parents. There may be mutual effects between fatherhood and motherhood. For example, the higher the mother’s satisfaction with the father’s involvement in parenting, the lower the mother’s parenting stress (39). The mutual affecting between fathers’ and mothers’ parenting experiences may also be a future research direction.

Third, the cross-sectional methodology used in this study cannot contribute to a definable cause-and-effect link. Experimental or longitudinal studies are required to determine the causal relationships. In particular, research has shown that parenting stress and marital quality interact in both directions (41, 47). In addition, the link between marital satisfaction and parenting burnout may show different directions in different contexts (76–78). Longitudinal surveys and research in various contexts are more appropriate research designs for the association between parenting and marriage. A qualitative study could also help to explore the developmental features and patterns of the PB phenomenon in greater depth (79). Further in-depth research requires the application of multiple methodologies.

## 5 Conclusion

This study found a positive correlation between parenting stress and PB among mothers in China. Perceived marital satisfaction acts as a partial mediating factor, and mothers’ SES may influence this mediating effect. Notably, the impact of parenting stress on marital satisfaction may be more pronounced for mothers with higher SES. These findings offer a deeper understanding of the role of marital satisfaction in alleviating PB among mothers in China. Moreover, findings provide a foundation for formulating more effective intervention strategies for PB, thereby promoting mothers’ well-being.

## Data Availability

The raw data supporting the conclusions of this article will be made available by the authors, without undue reservation.
